# Mentalizing abilities and serum lipid levels in adult MDD patients with childhood maltreatment – preliminary results

**DOI:** 10.1192/j.eurpsy.2022.393

**Published:** 2022-09-01

**Authors:** P. Nyarondi, Á. Péterfalvi, M. Szennai, N. Németh, T. Tényi, B. Czéh, M. Simon

**Affiliations:** 1 Medical School, University of Pécs, Department Of Psychiatry And Psychotherapy, Pécs, Hungary; 2 University of Pécs, Medical School, Pécs, Hungary, Department Of Laboratory Medicine, Pécs, Hungary; 3 Medical School, University of Pécs, Pécs, Hungary, Department Of Psychiatry And Psychotherapy, Pécs, Hungary; 4 Neurobiology of Stress Research Group, János Szentágothai Research Centre, University Of Pécs, Hungary, Pécs, Hungary; 5 Medical Faculty, University of Pécs, Hungary, Department Of Psychiatry And Psychotherapy, Pécs, Hungary; 6 János Szentágothai Research Centre, University of Pécs, Hungary, Neurobiology Of Stress Research Group, Pécs, Hungary

**Keywords:** major depressive disorder, childhood maltreatment, mentalizing, serum lipid levels

## Abstract

**Introduction:**

Childhood maltreatment (CM) contributes to negative mental and physical health outcomes including major depressive disorder (MDD), and an elevated risk for cardiovascular disease (CDV) in adults. Also, childhood maltreatment can be related to mentalizing deficits in MDD. Cardio-metabolic diseases often coincide with MDD and worsen its course and outcome. Little is known on the interplay of these factors.

**Objectives:**

We examined MDD patients with and without CM to explore the effects of CM on serum lipid and lipoprotein levels and assessed their mentalizing abilities. Self-oriented mentalizing was operationalized as emotional self-awareness/alexithymia, other-oriented mentalizing was defined as theory of mind (ToM).

**Methods:**

MDD patients (N=42) and healthy controls (n=20) matched in age, sex, and lifestyle were investigated. Total cholesterol, triglycerides, high- and low-density lipoproteins (HDL-C and LDL-C), body mass index, and exercise in a typical week were measured. Beck Depression Inventory, Childhood Trauma Questionnaire, Toronto Alexithymia scale, and the Reading the mind in the Eyes Test were used to assess clinical symptoms, mentalizing abilities and CM.

**Results:**

After controlling for depressive symptom severity, demographic and lifestyle variables, CM was found to be a strong predictor of serum lipid alterations. Mentalizing deficits correlated with CM. Serum triglycerides, HDL-C were significant predictors of ToM performance (P<0.05, and P=0.005) and alexithymia (P< 0.05, and P< 0.05) in the MDD group.

**Conclusions:**

Several, inter-correlated pathways may mediate the undesirable effects of CM on the course and outcome of MDD. According to our preliminary results, diminished self-awareness and ToM can be possible mediating factors.

**Disclosure:**

This work was financially supported by the Hungarian Brain Research Program (2017-1.2.1-NKP-2017-00002)

